# Predicting kidney graft function and failure among kidney transplant recipients

**DOI:** 10.1186/s12874-024-02445-6

**Published:** 2024-12-31

**Authors:** Yi Yao, Brad C. Astor, Wei Yang, Tom Greene, Liang Li

**Affiliations:** 1 Department of Biostatistics, University of Texas MD Anderson Cancer Center, Houston, TX USA; 2 School of Medicine and Public Health, University of Wisconsin-Madison, Madison, WI USA; 3 Perelman School of Medicine, University of Pennsylvania, Philadelphia, PA USA; 4 School of Medicine, University of Utah, Madison, UT USA

**Keywords:** Competing risk, Dynamic prediction, Graft failure, Kidney transplantation, Renal function

## Abstract

**Background:**

Graft loss is a major health concern for kidney transplant (KTx) recipients. It is of clinical interest to develop a prognostic model for both graft function, quantified by estimated glomerular filtration rate (eGFR), and the risk of graft failure. Additionally, the model should be dynamic in the sense that it adapts to accumulating longitudinal information, including time-varying at-risk population, predictor-outcome association, and clinical history. Finally, the model should also properly account for the competing risk by death with a functioning graft. A model with the features above is not yet available in the literature and is the focus of this research.

**Methods:**

We built and internally validated a prediction model on 3,893 patients from the Wisconsin Allograft Recipient Database (WisARD) who had a functioning graft 6 months after kidney transplantation. The landmark analysis approach was used to build a proof-of-concept dynamic prediction model to address the aforementioned methodological issues: the prediction of graft failure, accounted for competing risk of death, as well as the future eGFR value, are updated at each post-transplant time. We used 21 predictors including recipient characteristics, donor characteristics, transplant-related and post-transplant factors, longitudinal eGFR, hospitalization, and rejection history. A sensitivity analysis explored a less conservative variable selection rule that resulted in a more parsimonious model with reduced predictors.

**Results:**

For prediction up to the next 1 to 5 years, the model achieved high accuracy in predicting graft failure, with the AUC between 0.80 and 0.95, and moderately high accuracy in predicting eGFR, with the root mean squared error between 10 and 18 mL/min/1.73m2 and 70%-90% of predicted eGFR falling within 30% of the observed eGFR. The model demonstrated substantial accuracy improvement compared to a conventional prediction model that used only baseline predictors.

**Conclusion:**

The model outperformed conventional prediction model that used only baseline predictors. It is a useful tool for patient counseling and clinical management of KTx and is currently available as a web app.

**Supplementary Information:**

The online version contains supplementary material available at 10.1186/s12874-024-02445-6.

## Background

Kidney transplantation (KTx) and hemodialysis are two commonly used treatments for patients with end-stage kidney disease (ESKD). The former provides significantly better quality of life and life expectancy than the latter. Nonetheless, patients receiving KTx are at risk of losing their grafts, making it important to monitor graft health on a regular basis. This includes assessment of graft function, often quantified by the estimated glomerular filtration rate (eGFR), and the risk of graft failure. The risk of decreased eGFR and graft failure are used clinically to make decisions on specific treatments, including intensity of immunosuppressive therapy. It is therefore of substantial clinical importance to develop prediction models for graft health with data routinely available from clinical practice. Such prognostic tools are useful for patient counseling and improving preventive care.

Many risk prediction models have been developed among KTx recipients [1–10]. However, there remain important gaps in this research. Most studies focus on the prediction of graft failure or graft survival [3]. The latter is defined as the time to graft failure or death. Patients with a functioning allograft can live for many years. During most of this time, the risk of graft failure is not excessively high, and the patient and physician may be more interested in graft function. Therefore, it is useful to have a model that predicts both the risk of graft failure within the next $\Delta$ years ($\Delta$ is a pre-specified time window, called the prediction horizon) and, if graft failure does not occur during this time window, the future eGFR. In this paper, we call it the prediction of graft health, which includes a concurrent prediction of both graft function (i.e., eGFR) and graft failure. To our knowledge, such a concurrent prediction model has not been developed in the literature, and it is one of the main innovations of this paper.

A related issue to the prediction of graft health is the proper adjustment for the competing risk of death, which has been overlooked in many graft failure risk prediction efforts. Death precludes the subsequent occurrence of graft failure, and not all deaths are directly related to kidney disease. Treating death as censoring in a survival regression analysis causes bias in estimating the probability of graft failure [11–14] because censoring implies that graft failure can occur after the censoring event, despite not being observed in the data. This is not the case when the censoring event is death. Analyzing the composite event of death and graft failure (i.e., graft survival) may result in decreased prediction accuracy if the predictors are selected to be risk factors of kidney disease but all-cause death is used. Deaths due specifically to graft dysfunction are often not indicated in the available data sources, and they can also be difficult to define clinically. In this paper, we used a competing risk regression model to analyze graft failure and all-cause death.

Many graft failure models are designed for prediction at a specified time point, such as pre-transplant [1, 10], right after surgery [1, 4], 1 year after the surgery [1, 15], or at the time of a specific events, such as post-transplant biopsy [5]. In this paper, we propose a model that can predict at any time during post-transplantation, using the cumulative prognostic information up to the time of prediction. Since those with high graft failure risk tend to have the event early, the at-risk population (i.e., those alive with a functioning graft, to whom the model is applicable) varies over time, as do the predictor-outcome associations [1]. This kind of model can be fit using dynamic prediction [16–18]. This statistical technique has recently been used to predict graft survival among pediatric [19] and adult [20] patients. However, these studies used a single longitudinal prognostic biomarker (serum creatinine) due to computational feasibility and software availability of the statistical method. In this paper, we used a different method of dynamic prediction that works with multiple longitudinal predictors with tractable computation, and it achieves a concurrent prediction of graft health instead of graft survival only.

## Materials and methods

### Study cohort

Our data source is the Wisconsin Allograft Recipient Database (WisARD), which was initiated in 1984 to collect information on all solid organ transplants performed at the University of Wisconsin (UW), using a kerncombination of electronic health records (EHR) and active protocolized data collection. The dataset used in this study included 3,893 graft recipients who received a kidney transplant between 1994 and 2013 and were followed for a median period of 67.3 months (range 6–232 months) from baseline, defined as the 6th month after transplantation throughout this paper. At the data extraction in 2019, 532 (13.7%) recipients had graft failure, 774 (19.9%) had died with a functioning graft, and the rest 2,587 (68.4%) were survivors with a functioning graft. The median of observed follow-up times from baseline were 54.5 and 65.3, and 71.2 months for patients experiencing graft failure, death and for survivors, respectively. Recipients had frequent measurements of eGFR during the follow-up period, ranging from 1 to 567 measurements, and 50% had more than 60 measurements. The times of these measurements were unsynchronized among different graft recipients.

This research has been approved by the Institutional Review Board of the participating institutions. The authors abided by guidelines laid out by the Declaration of Helsinki. The clinical and research activities being reported are consistent with the Principles of the Declaration of Istanbul as outlined in the “Declaration of Istanbul on Organ Trafficking and Transplant Tourism”. Transplant coordinators review all available pre-transplant, peri-operative, and post-operative records, confirm the information, and enter data. All post-discharge clinic visits and hospitalizations include ascertainment of any intervening issues, events or hospitalizations. For encounters outside UW, the coordinators request records from the local physician or hospital and review and enter available information. All clinical, laboratory, and biopsy data are collected from HealthLink, the UW’s EHR system, and any other sources and reviewed by the coordinator before entry. A Delinquent Lab List, listing patients and results that have not been entered for 3 months, is generated monthly to prompt coordinators to actively pursue lab results and other data. Serum creatinine is routinely measured monthly post-transplant. Only outpatient labs were used in these analyses.

### Outcomes

We evaluated two time-to-event outcomes: the times from baseline to graft failure and to all-cause death with a functioning graft. For survivors with a functioning graft, their time-to-event outcomes were censored at the end of follow-up. Graft failure was defined as re-transplantation or return to dialysis. Death after graft failure was not used in the analysis because a patient’s health condition and disease management change substantially after graft failure. The longitudinally measured eGFRs served as both predictor and outcome variables. The eGFR data before the prediction time were used to predict the future eGFR trajectory. The eGFR was calculated from the CKD-EPI equation [21].

### Candidate predictors

We identified time-invariant (baseline) and time-varying (longitudinal) predictors based on published literature on risk prediction for graft failure and graft function. The time-invariant predictors include pre-transplant recipient and donor characteristics, transplant-related risk factors, and post-transplant risk factors (Table 1). The time-varying predictors include eGFR, number of hospitalization and rejection episodes in the past 12 months. The eGFR was measured frequently in WisARD. To avoid excessive weighting of patients with frequent measurements, we retained at most one eGFR measurement per recipient and per month since baseline, by choosing the measurement closest to the mid-point of the month. Since the dynamic prediction model enables the prediction to be made at any time after the baseline, we extracted numerical features of the longitudinal eGFR data (slope and volatility in the past two years), and recurrent hospitalization and rejection episode data within the year prior to the prediction time. We used these features as additional predictors. There are sporadic missing data (Table 1) in the months of pre-transplant dialysis (0.24%), Human Leukocyte Antigens mismatches (0.02%), peak Panel-Reactive Antibody (0.1%), donor hypertension (9.7%), donor diabetes (11%), donor body mass index (1.82%), beta-2-microglobulin (0.53%), calcium (1.77%), magnesium (2.03%), and phosphorus (1.77%). These are imputed with mean, median or mode as appropriate.

**Table 1:** Baseline characteristics of 3,893 kidney transplant recipients with a functioning graft at the 6th month post-transplant. The data are summarized for all the subjects, and also by the types of outcomes. Continuous variables are presented as mean (standard deviation). Categorical variables are presented as count (%). Non-normal and count variables are presented as median [25% quantile; 75% quantile]

Outcome	Total	Censored survivors	Graft failure	Death
N	3,893	2,587	532	774
**Pre-transplant Recipient Characteristics**				
Age at Tx (year)	51.1 (12.9)	50.1 (12.8)	47.3 (13.5)	57.0 (10.7)
Female	1,535 (39.4%)	1,037 (40.1%)	211 (39.7%)	287 (37.1%)
Black race	377 (9.68%)	237 (9.16%)	87 (16.4%)	53 (6.85%)
Having prior Tx	848 (21.8%)	527 (20.4%)	127 (23.9%)	194 (25.1%)
Pre-Tx HD (months)	14 [1; 34]	12 [0; 32]	19 [8; 40]	17 [5; 34]
ESRD				
DM	978 (25.1%)	514 (19.9%)	156 (29.3%)	308 (39.8%)
HTN	432 (11.1%)	286 (11.1%)	65 (12.2%)	81 (10.5%)
PKD	529 (13.6%)	425 (16.4%)	44 (8.27%)	60 (7.75%)
GN	966 (24.8%)	696 (26.9%)	145 (27.3%)	125 (16.1%)
Use of maintenance IMS (Yes)				
Tac	2,671 (68.6%)	2,030 (78.5%)	277 (52.1%)	364 (47.0%)
CsA	927 (23.8%)	419 (16.2%)	177 (33.3%)	331 (42.8%)
Use of Induction therapy (Yes)				
Alemtuzumab	736 (18.9%)	368 (14.2%)	150 (28.2%)	218 (28.2%)
IL2	2,175 (55.9%)	1,555 (60.1%)	238 (44.7%)	382 (49.4%)
ATG (Thymo)	815 (20.9%)	541 (20.9%)	126 (23.7%)	148 (19.1%)
**Donor Characteristics**				
Living donor	1,528 (39.2%)	1,101 (42.6%)	164 (30.8%)	263 (34.0%)
Age (years)	43.2 (14.6)	42.1 (14.4)	45.3 (14.1)	45.4 (15.0)
Female	1,807 (46.4%)	1,199 (46.3%)	244 (45.9%)	364 (47.0%)
Black race	290 (7.45%)	198 (7.65%)	48 (9.02%)	44 (5.68%)
BMI (kg/${\text{m}}^{2}$)	27.7 (6.53)	27.7 (6.55)	27.9 (6.84)	27.7 (6.23)
DM	180 (4.62%)	94 (3.63%)	47 (8.83%)	39 (5.04%)
COD				
Anoxia	654 (16.8%)	476 (18.4%)	76 (14.3%)	102 (13.2%)
CVD	810 (20.8%)	426 (16.5%)	162 (30.5%)	222 (28.7%)
Trauma	800 (20.5%)	529 (20.4%)	110 (20.7%)	161 (20.8%)
Tumor	20 (0.51%)	7 (0.27%)	5 (0.94%)	8 (1.03%)
KDPI	45.5 (26.6)	41.4 (25.5)	53.3 (27.0)	51.8 (27.2)
**Transplant-Related and Post-transplant Factors**				
HLA mismatches	4 [3; 5]	4 [3; 5]	4 [3; 5]	4 [2; 5]
Peak PRA ≥ 1	1,595 (41.0%)	1,045 (40.4%)	237 (44.5%)	313 (40.4%)
DGF	678 (17.4%)	372 (14.4%)	140 (26.3%)	166 (21.4%)
eGFR (mL/min/1.73${\text{m}}^{2}$)^a^	55.6 (18.6)	57.7 (18.1)	48.1 (18.6)	53.4 (18.4)
B2M (mcg/L)^b^	1.29 (0.49)	1.25 (0.49)	1.41 (0.52)	1.35 (0.46)
Mg (mq/dL)^b^	1.79 (0.29)	1.76 (0.29)	1.86 (0.32)	1.83 (0.29)
Ca (mg/dL)^b^	9.43 (0.68)	9.44 (0.66)	9.45 (0.71)	9.39 (0.70)
Ph (mg/dL)^b^	2.75 (0.88)	2.73 (0.84)	2.80 (0.99)	2.78 (0.90)
Hospitalization^c^	1,521 (39.1%)	883 (34.1%)	263 (49.4%)	375 (48.4%)
Rejection^c^	657 (16.9%)	324 (12.5%)	165 (31.0%)	168 (21.7%)

Abbreviations in this table and also in other tables and figures of this paper: *eGFR* estimated glomerular filtration rate, *Tx* kidney transplantation, *HD* hemodialysis, *ESRD* end-stage renal disease (for renal transplant recipients), *DM* diabetes mellitus, *HTN* hypertension, *PKD* polycystic kidney disease, *GN* glomerulonephritis, *ESRD:DM* ESRD caused by diabetes mellitus (similarly for ESRD:HTN, ESRD:PKD, and ESRD:GN), *IMS* immunosuppressant, *Tac* Tacrolimus, *CsA* Cyclosporin A, *IL2* Interleukin 2, *ATG* anti-thymocyte immunoglobulin (also called thymoglobulin or Thymo), *BMI* body mass index, *COD* cause of death (for deceased donors), *KDPI* kidney donor profile index (calculated for deceased donor only and is 0 for living donor), *HLA* human leukocyte antigens, *PRA* panel-reactive antibody, *DGF* delayed graft failure (occurs in the first week of kidney transplantation), *B2M* beta-2-microglobulin, *Mg* magnesium, *Ca* calcium, *Ph* phosphorus

^a^The eGFR measurement closest to the 6 months after transplantation

^b^The first measurement of the laboratory results within the first 6 months after transplantation

^c^Any hospitalization or rejection event within the first 6 months after transplantation

### Prediction model development

Our model development is based on dynamic prediction with landmark modeling. We extended our previous work on landmark prediction modeling for clinical events with censoring [22, 23] and competing risks [24, 25] by incorporating an additional model for predicting eGFR. The landmark modeling allowed prediction made at any time after 6 months post-transplant, called the landmark time. The model corresponding to each landmark time was fitted among all the “at-risk” recipients at that time, i.e., those who lived beyond the landmark time without graft failure. Restriction to the at-risk recipients was necessary because this was the population to whom the prediction was applicable. This population varied over time as those with higher risk of graft failure or death were more likely to reach these events and terminate follow-up. Landmark modeling was adaptive to change in the applicable population of its prediction, a distinct feature of dynamic prediction.

In a nutshell, the models at each landmark time include a pair of cause-specific Cox proportional hazard (CSH) models for graft failure and death [26], and a generalized estimating equations (GEE) model fitted to the longitudinal eGFR data measured between the prediction time and the prediction horizon. The predicted probabilities of graft failure and death and the predicted eGFR at the horizon were calculated directly from the fitted models. All three sub-models used time-invariant and time-varying predictors at the landmark time. Backward model selection was used to select a parsimonious model from the initial set of time-invariant and time-varying predictors. For comparison with dynamic prediction, we also fitted a static prediction model and compared it to the dynamic prediction results. The static prediction model (SPM) is widely used in medical literature [22, 23, 27]. It uses baseline predictors to predict the time from baseline to graft failure and death and post-baseline eGFR data. The model was fitted to all the patients at baseline (i.e., 6 months post-transplantation, in our context). Further details of model fitting are elaborated in Section S2 of the online supplementary material.

### Prediction accuracy assessment

We assessed the prediction accuracy through cross-validation. We randomly split WisARD recipients into three subsets of approximately equal sizes. We fit the dynamic prediction model using two subsets and tested the prediction accuracy in the third. The results were averaged over three choices of the testing subset to form the prediction accuracy measures from a split. To further reduce random variation, we repeated the above split 5 times, and reported the average prediction accuracy results in this paper.

The prediction accuracy measures for graft failure and death included the area under time-dependent receiver operating characteristic curve (AUC) and time-dependent Brier score (BS) for competing risks [24]. We resample the training set 500 times within each cross-validation and compute the increments in prediction accuracy of the competing risk model between the proposed LM and benchmark SPM in each resampling. If the 95% bootstrap confidence interval contains zero, then the difference is validated. The prediction accuracy for eGFR was the root mean squared error (RMSE) between the predicted eGFR at the horizon and observed eGFR within two months of the horizon. We also calculated the proportion of patients that the predicted eGFR fall within 30% (P30) and 50% (P50) of the observed eGFR, an alternative measure that is often used in chronic kidney disease research [28]. Higher AUC, lower BS, lower RMSE, and higher P30 and P50 indicate better prediction performance. We considered 3 prediction horizons at 1 year, 3 year, and 5 years.

## Results

### Baseline and time-varying characteristics of patients

Table 1 shows the baseline characteristics of all 3,893 patients overall and by outcome (death cases, graft failure cases, censored survivor cases). The mean age at transplantation was 51.1 years. The age was older among the death cases (57.0 years). There were nearly 40% female and 10% black recipients. Approximately 22% of recipients had a prior transplantation. The recipients had a median of 14 months of hemodialysis before transplantation, with notably longer hemodialysis history (19 months) among the graft failure cases. Approximately 40% of recipients received a kidney graft from a living donor. Compared with the censored survivors, the graft failure cases and death cases had notably lower proportion of living donors (30.8%, 34.0% vs. 42.6%), older donor ages (45.3, 45.4 vs. 42.1 years), higher KDPI (53.3, 51.8 vs. 41.4), higher proportion with hospitalization (49.4%, 48.4% vs. 34.1%) or rejection events (31.0%, 21.7% vs. 12.5%) within the first 6 months after transplantation.

Table 2 shows the time-varying characteristics of the recipients who remained at-risk at each of the six landmark times. As time progresses, the sample size decreases because of attrition. While these recipients survived each landmark time without graft failure, their risks of hospitalization and rejection increased. The age increased as expected. Because of attrition, the difference in mean age between consecutive landmark times does not equal to the difference in landmark times. While the eGFR often declined over time at the individual level, those with lower eGFR were at increased risk of graft failure and dropped out from the at-risk patients. These two opposite trends resulted in stable mean eGFR levels across the landmark times.

**Table 2:** Time-varying predictors of renal transplant recipients who were at-risk by the landmark time of 12, 24, 36, 48, and 60 months post-transplantation

Months after Tx	6	12	24	36	48	60
**Number of patients**	**3,893**	**3,154**	**2,525**	**2,012**	**1,668**	**1,381**
Age (years)	51.1 (12.9)	52.4 (12.8)	53.5 (12.6)	54.6 (12.6)	55.1 (12.6)	56.3 (12.4)
eGFR (ml/min/1.73${\text{m}}^{2}$)	55.6 (18.6)	54.9 (18.6)	56.9 (19.8)	56.6 (20.2)	56.1 (19.8)	55.8 (19.9)
Hospitalization^a^	1,521 (39.1%)	1,389 (44.0%)	1,255 (49.7%)	1,055 (52.4%)	933 (55.9%)	812 (58.8%)
Rejection event^a^	657 (16.9%)	615 (19.5%)	537 (21.3%)	439 (21.8%)	365 (21.9%)	304 (22.0%)

^a^Within the last 12 months of the landmark time

### Estimated model parameters

The model selection procedure identified 21 predictors in the final model, 3 of which were time-varying. Since the estimated model coefficients vary with landmark time, we plotted their relationship as curves in Figures S1 and S2. We further presented details of the coefficients and their 95% confidence intervals (CIs) at two selected landmark times in Table S1. Younger age, hospitalization, rejection, donor DM, recipient ESRD due to DM, lower eGFR, increasing HLA mismatches, higher KDPI, longer pre-Tx hemodialysis, and prior Tx are statistically significantly associated with higher risk of graft failure, as their 95% CIs do not cover zero at most of the landmark times (Figure S1). Older age, hospitalization, rejection, recipient ESRD due to DM or HTN, higher B2M, longer pre-Tx hemodialysis and prior Tx are statistically significantly associated with higher mortality (Figure S1). Hospitalization, rejection, older donor, and higher B2M are statistically significantly associated with lower eGFR at the horizon; higher eGFR at the landmark time is associated with higher eGFR at the horizon, as expected (Figure S2). History of hospitalization and rejection have persistently strong and significant associations with subsequent graft failure, death and eGFR outcomes, but they are rarely studied in conventional static prediction modeling. This result exemplifies the benefit of dynamic prediction. Some predictor-outcome associations vary with the landmark time. The most notable example is eGFR in the graft failure model, where the eGFR becomes more predictive as the recipient lived longer with the graft, while the KDPI, based on pre-transplant information, appears less predictive (Figure S1).

### Prediction accuracy

**Fig. 1:**
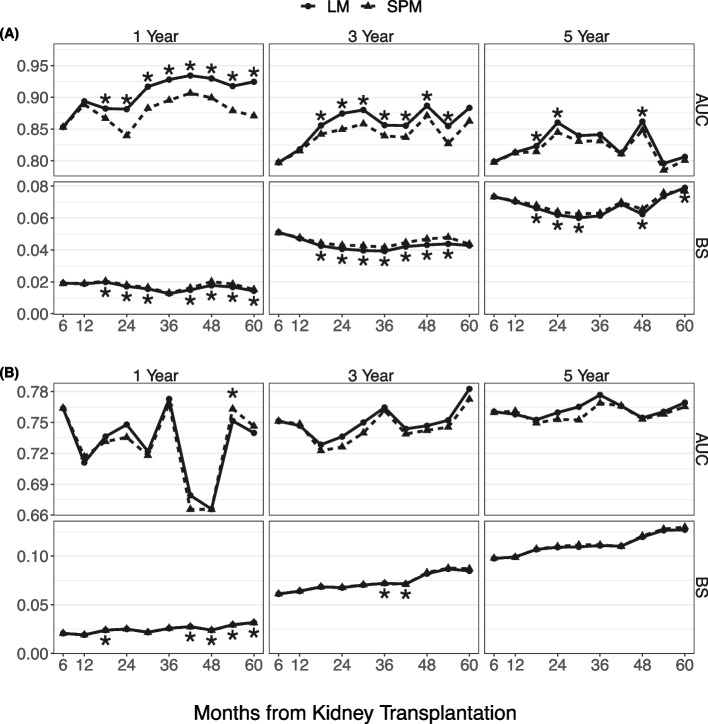
The time-dependent AUC and BS of LM and SPM in predicting competing risks. Legend: The AUC and BS values vary with prediction time (ranging from 6 to 60 months post-transplant), and for three prediction horizons (1, 3, 5 years). The asterisk (*) indicates that the difference in AUC or BS between LM and SPM is statistically significant as the 95% bootstrap confidence interval does not cover zero. **A** graft failure; **B** death

Figure 1 displays cross-validated time-dependent AUC and BS for the graft failure and death outcomes. Since the model allows a prediction to be made at any month between the 6th and 60th months after transplantation, the estimated AUC and BS are presented as curves demonstrating how the prediction accuracy vary with the prediction time. We presented AUC and BS for a variety of prediction horizons. The landmark model shows high predictive accuracy, especially for graft failure. The AUC for graft failure is approximately between 0.80 and 0.95. The AUC for death is approximately between 0.72 and 0.78. Most predictors are related to kidney transplant and graft function, and hence they have stronger prognostic association with graft failure than with all-cause death. As expected, the AUC decreases and BS increases with a longer prediction horizon, because it is more difficult to predict outcomes over a longer period. In comparison with SPM, the dynamic prediction model shows consistently better performance in predicting graft failure in next 1 and 3 year at prediction times at or after 18 months post-transplantation. The predictive performance for death with a functioning graft is similar between the two models. The AUC and BS of the SPM are the best when the prediction time is close to the baseline and deteriorate as over time, because the at-risk population increasingly deviated from the baseline population, from which the SPM was developed. This result demonstrates the importance for the prediction model to be adaptive to the changing at-risk population.

**Fig. 2:**
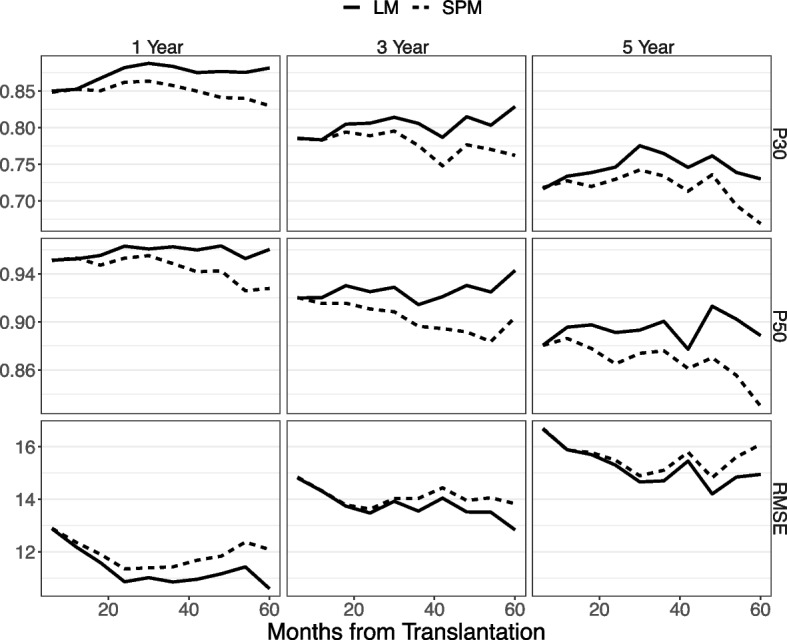
Comparison of landmark GEE model with static GEE model in predicting the future Egfr. Legend: Predictive accuracy metrics for eGFR include the proportion that the predicted values fall within 30% (P30) and 50% (P50) of observed eGFR values at the prediction horizon, and the root mean squared error (RMSE, mL/min/1.73m^2^) between the predicted and observed eGFR. The results are displayed by prediction horizon (1, 3, 5 years) and the prediction time ranges from 6 to 60 months post-transplant

Figure 2 shows three prediction accuracy measures of eGFR, presented by prediction horizon and landmark time. As expected, the prediction accuracy decreases with larger prediction horizon. The dynamic prediction model clearly outperforms the SPM in all scenarios. The SPM performance deteriorates as the at-risk population moves away from the baseline. The P30 and P50 of the dynamic prediction model are generally high, e.g., 70%−90% of predicted eGFR falling within 30% of the observed eGFR. This suggests good accuracy, especially for shorter horizons. The RMSE in Fig. 2 is in the range of 11–16 mL/min/1.73m2. This is close to the measurement error variance of eGFR (see Section S3 of Supplementary Material). The measurement error of eGFR reflects the physiological and bioassay variability. It may serve as a lower bound for the RMSE that an eGFR prediction model can achieve.

## Discussion

While short-term outcomes following KTx are excellent, with more than 95% of grafts and recipients surviving beyond one year, long-term graft and patient survival remain suboptimal [29]. Long-term management of KTx recipients requires a constant balance of maintaining adequate immunosuppression to prevent graft rejection and potential graft loss weighed against over-immunosuppression and the concomitant higher risks of toxicity, including graft dysfunction and death due to infection or malignancy. Identification of recipients at highest risk is needed to inform clinical practice decisions to prevent these outcomes and extend graft and recipient survival. Preventing long-term graft failure has substantial benefits both for the individual recipient and for patients remaining on the lengthening waiting list.

To the best of our knowledge, the model developed in this manuscript is the first to jointly predict both the risk of graft failure and graft function. Death is modeled as a competing risk event. Such a comprehensive prediction of the overall graft health, includes both graft failure and graft function while also accounting for death, is likely to be useful for efforts to improve patient-centered care and personalized treatment. Our dynamic prediction model was developed using all longitudinal data. Compared with the static prediction model, which uses only baseline and outcome data, the dynamic prediction model has demonstrated notably improved prediction accuracy. It is also scientifically better justified because the model is adaptive to the time-varying at-risk population, time-varying predictor effects, and longitudinal history.

Variable selection is challenging in a dynamic prediction model, and to our knowledge, it remains an unexplored topic in the statistical methodological literature. We have used a conservative variable selection rule in this paper that resulted in 21 predictors. While this approach reduces the chance of missing important predictors and can be used to demonstrate that our proposed landmark model can accommodate relatively more time-invariant and time-varying predictors with tractable computation in comparison with the joint modeling counterparts to dynamic prediction, a large model may be inconvenient to use in practice. In Section S4, we explored a less conservative variable selection rule that resulted in a more parsimonious model with fewer predictors but comparable prediction accuracy. Future statistical research is needed to develop data-driven variable selection methods for dynamic prediction problems.

We are not the first to use dynamic prediction model in KTx research. Kaboré et al. [19] and Fournier et al. [20] used this statistical technique to predict graft survival among pediatric and adult patients. However, their approach was based on joint modeling, i.e., estimating a joint distribution of longitudinal eGFR data and time to graft failure. This approach cannot accommodate the three longitudinal variables in our problem: eGFR, hospitalization and rejection events. The latter two are longitudinal recurrent event data. There were significant changes over time in the at-risk population and in the proportion of those remaining at-risk with a history of hospitalization and rejection in our study (Table 2). Our dynamic prediction model is based on the landmark analysis, which can accommodate relatively more time-invariant and time-varying predictors with efficient and tractable computation, in contrast to their joint modeling counterparts for dynamic prediction problems.

The previous approaches also did not account for the competing risk by death. As the number of deaths in WisARD was greater than the number of graft failures, this is a significant advantage of our approach. Further, there are some complex relationships between the predictors of each of these outcomes which may be masked if this competing risk is ignored. For example, younger individuals generally have stronger immune systems and younger age is known to be associated with greater risk of graft rejection, whereas younger recipients also have a significantly lower risk of death.

Despite these advantages, this study also has several limitations. First, the prediction accuracy came from internal cross-validation instead of external validation. Prediction model development usually includes three steps: model development and internal validation with the training dataset, external validation with an independent dataset, and evaluation in a prospective study. External validation will be our next step of work. Second, we noted that the landmark approach might not be the best option for future eGFR prediction since it does not learn from the entire individual-level eGFR trajectories. Third, our model was evaluated for predictions made up to 60 months after transplantation. The 60-month time period was chosen to ensure that there are enough follow-up and outcome events for a prediction horizon of up to 5 years in internal cross-validation. Fourth, we performed limited exploration of the predictive effect of eGFR history by using certain prespecified and interpretable features of the longitudinal eGFR history, such as the estimated subject-specific slopes and variances from the past eGFR values, and they were not found to be significantly associated with the future eGFR when the “current” eGFR at the time of prediction is in the GEE model. It is worthwhile to consider alternative models for eGFR trajectory and study whether the prediction of eGFR can be further improved by using the longitudinal history more effectively.

A web application is available for the model in this paper (https://yyao3.shinyapps.io/ktxdynpred/). Users can enter the predictors, and the web application produces predicted probabilities and eGFR at prespecified horizon.

This paper uses a new landmark dynamic prediction model to simultaneously predict the risk of graft failure, death, and future renal function among patients receiving post KTx care. The model effectively uses both time-invariant and time-varying predictors and it outperformed conventional prediction model that used only baseline predictors in our dataset. It is a useful tool for patient counseling and clinical management of KTx.

## Supplementary Information

Supplementary Material 1.

## Data Availability

Requests for de-identified data from WisARD should be directed to Dr. Astor at bcastor@medicine.wisc.edu.
