# Supplementary material for: Predicting kidney graft function and failure among kidney transplant recipients
Source: BMC Med Res Methodol. 2024 Dec 31;24:324. doi: 10.1186/s12874-024-02445-6 (PMC11687162; doi:10.1186/s12874-024-02445-6)
Supplement: Supplementary file 1 — Supplementary Material 1. [file 12874_2024_2445_MOESM1_ESM.pdf]

Supplementary Materials for

"Predicting kidney graft function and failure among kidney transplant recipients"

by Yi Yao, Brad Astor, Wei Yang, Tom Greene, and Liang Li

Table of Contents

Section S1. Additional Tables and Figures, including Table S1 and Figures S1, S2, S3 and S4.
Section S2. Technical details of the statistical analysis
Section S3. An approximation to the measurement error variance of eGFR
Section S4. Developing a parsimonious landmark model with reduced sets of predictors and comparing its prediction performance with the static model, with Figures S5 and S6.

Section S1. Additional Tables and Figures

Table S1. Estimated regression coefficients and 95% confidence intervals (CIs) for the landmark models at 6 and 36 months after Tx. Each landmark model includes three sub-models: two cause-specific hazard (CSH) models for graft failure and death and one GEE model for eGFR. The GEE model parameters depend on the horizon. Abbreviations are similar to Table 1.

**Covariates**	**CSH (HR [95% CI])**		**GEE (Coefficient [95% CI])**	
	**Graft failure**	**Death**	**1-Year**	**3-Year**
	**At baseline (6 months post-transplantation)**			
HLA mismatches	1.12 [1.05, 1.19]	0.99 [0.94, 1.04]	-0.12 [-0.25, 0.01]	-0.1 [-0.26, 0.07]
Prior Tx	1.18 [0.92, 1.52]	1.58 [1.27, 1.97]	-0.38 [-1, 0.25]	-0.93 [-1.71, -0.16]
Pre Tx HD	1 [1, 1.01]	1 [1, 1.01]	0 [0, 0.01]	-0.01 [-0.02, 0]
KDPI	1.01 [1, 1.01]	1 [1, 1.01]	0 [-0.01, 0.01]	0.01 [0, 0.02]
Peak PRA	1.21 [0.99, 1.49]	0.96 [0.8, 1.13]	-0.17 [-0.62, 0.29]	-0.17 [-0.72, 0.39]
Age	0.97 [0.96, 0.98]	1.06 [1.05, 1.07]	0.01 [-0.01, 0.03]	0.04 [0.01, 0.06]
eGFR	0.98 [0.97, 0.98]	1 [1, 1.01]	0.8 [0.78, 0.83]	0.78 [0.75, 0.8]
Hospitalization†	3.61 [2.65, 4.92]	1.89 [1.54, 2.32]	-2.35 [-2.8, -1.9]	-3.39 [-3.95, -2.82]
Rejection†	1.36 [1.1, 1.68]	1.51 [1.23, 1.87]	-1.04 [-1.62, -0.46]	-0.6 [-1.34, 0.15]
B2M	1.35 [1.09, 1.67]	1.68 [1.41, 1.99]	-2.2 [-2.71, -1.68]	-2.22 [-2.86, -1.57]
Mg	1.54 [1.1, 2.16]	1.25 [0.95, 1.65]	-1.55 [-2.35, -0.75]	0.36 [-0.63, 1.34]
ESRD:DM	1.67 [1.34, 2.08]	1.9 [1.6, 2.25]	0.23 [-0.28, 0.74]	-0.23 [-0.89, 0.42]
ESRD:HTN	1.45 [1.07, 1.96]	1.29 [1, 1.67]	-0.31 [-1.02, 0.4]	0.52 [-0.42, 1.47]
Thymo	1.06 [0.57, 1.99]	1.27 [0.78, 2.09]	-0.54 [-1.56, 0.49]	-1.59 [-2.93, -0.25]
IL2	1.11 [0.61, 2.02]	1.15 [0.72, 1.81]	-0.71 [-1.61, 0.19]	-0.94 [-2.08, 0.19]
Alemtuzumab	1.18 [0.64, 2.16]	1.37 [0.85, 2.2]	-0.46 [-1.43, 0.51]	-1.24 [-2.42, -0.07]
Tac	0.93 [0.71, 1.23]	1.07 [0.82, 1.4]	1.24 [0.48, 2]	1.45 [0.45, 2.45]
CsA	1 [0.75, 1.34]	1.38 [1.06, 1.81]	0.07 [-0.7, 0.85]	-0.39 [-1.38, 0.61]
Donor:Age	1.01 [1, 1.02]	1 [1, 1.01]	-0.12 [-0.14, -0.1]	-0.18 [-0.21, -0.16]
Donor:BMI	0.99 [0.97, 1.01]	0.99 [0.98, 1]	0.03 [0, 0.07]	-0.05 [-0.09, 0]
Donor:DM	1.69 [1.18, 2.42]	1.13 [0.8, 1.6]	-2.37 [-3.28, -1.46]	-3.3 [-4.3, -2.3]
Time gap*	---	---	0.4 [0.28, 0.52]	0.23 [0.18, 0.28]
	**At 36 months post-transplantation**			
HLA mismatches	1.12 [1.02, 1.23]	0.99 [0.94, 1.06]	0.09 [-0.26, 0.44]	-0.32 [-0.54, -0.1]
Prior Tx	1.21 [0.85, 1.73]	1.54 [1.15, 2.06]	-0.56 [-1.67, 0.55]	-0.78 [-1.95, 0.39]
Pre Tx HD	1 [1, 1.01]	1 [1, 1.01]	-0.01 [-0.02, 0.01]	-0.01 [-0.02, 0.01]
KDPI	1.01 [1, 1.01]	1 [1, 1]	-0.02 [-0.04, 0.01]	-0.01 [-0.02, 0.01]
Peak PRA	1.21 [0.91, 1.62]	1.08 [0.86, 1.34]	0.94 [-0.33, 2.21]	-0.15 [-0.99, 0.68]

Covariates	CSH (HR [95% CI])		GEE (Coefficient [95% CI])	
	Graft failure	Death	1-Year	3-Year
Age	0.98 [0.97, 0.99]	1.06 [1.05, 1.07]	0 [-0.03, 0.03]	-0.01 [-0.04, 0.03]
eGFR	0.95 [0.94, 0.96]	0.99 [0.99, 1]	0.96 [0.83, 1.08]	0.89 [0.81, 0.96]
Hospitalization‡	3.1 [1.94, 4.96]	2.03 [1.54, 2.69]	-1.15 [-1.9, -0.41]	-1.96 [-2.78, -1.15]
Rejection‡	3.59 [2.33, 5.51]	1.84 [1.1, 3.1]	-5.57 [-7.35, -3.8]	-6.08 [-9.18, -2.99]
B2M	1.14 [0.83, 1.58]	1.69 [1.33, 2.14]	-1.44 [-2.28, -0.6]	-1.46 [-2.49, -0.43]
Mg	1 [0.64, 1.57]	1.43 [1, 2.05]	-0.08 [-1.83, 1.66]	-0.82 [-2.16, 0.52]
ESRD:DM	1.82 [1.32, 2.5]	1.9 [1.52, 2.36]	-0.06 [-1.05, 0.92]	-0.27 [-1.22, 0.68]
ESRD:HTN	1.31 [0.83, 2.06]	1.44 [1.05, 1.98]	-1.42 [-2.93, 0.09]	0.43 [-1.01, 1.87]
Thymo	1.28 [0.52, 3.14]	1.28 [0.68, 2.4]	-1.21 [-2.84, 0.42]	-1.59 [-3.34, 0.15]
IL2	1.33 [0.57, 3.12]	1.23 [0.69, 2.2]	0.38 [-0.9, 1.65]	-0.36 [-1.77, 1.05]
Alemtuzumab	1.41 [0.59, 3.36]	1.33 [0.73, 2.42]	0.92 [-0.66, 2.51]	1.1 [-0.53, 2.72]
Tac	0.79 [0.54, 1.16]	0.98 [0.7, 1.38]	-0.94 [-2.79, 0.91]	0.77 [-1.1, 2.64]
CsA	0.9 [0.61, 1.35]	1.27 [0.92, 1.75]	-1.45 [-3.63, 0.72]	-0.85 [-2.86, 1.15]
Donor:Age	1 [0.99, 1.01]	1 [0.99, 1.01]	-0.08 [-0.12, -0.05]	-0.14 [-0.17, -0.1]
Donor:BMI	1.01 [0.98, 1.03]	1 [0.98, 1.01]	-0.06 [-0.12, 0.01]	-0.03 [-0.1, 0.05]
Donor:DM	1.72 [0.99, 2.97]	1.33 [0.82, 2.14]	-1.39 [-3.07, 0.29]	-0.05 [-1.9, 1.79]
Time gap*	---	---	0.16 [-0.04, 0.37]	0.03 [-0.03, 0.09]

† Within the first 6 months after transplantation. ‡ Within the last 12 months of the landmark time.
* Time gap is the difference between the measurement time and the landmark time. This variable is only defined for GEE model.

The following are figure legends for Figures S1, S2, S3 and S4. High-resolution images of these figures are provided at the end of this document

Figure S1. Estimated regression coefficients of the cause-specific hazard (CSH) models for graft failure (A) and death (B). The coefficients are log cause-specific hazard ratios (solid line) with 95% confidence intervals (gray band). They are displayed as functions of the landmark time. The red dashed horizontal line is the reference line at zero. Abbreviations are similar to Table 1 in the paper.

Figure S2. Estimated regression coefficients of the GEE models for predicting eGFR at 1 year horizon (red) or 3 year horizon (cyan). The coefficients displayed as solid line, with 95% confidence intervals in colored band. The model parameters are displayed as functions of the landmark time. The red dashed horizontal line is the reference line at zero. Abbreviations are similar to Table 1.

Figure S3: **Illustration of the landmark prediction framework. (A) A hypothetical dataset where the longitudinal measurements (triangles) follow a synchronized time grid.** The six subjects who had longitudinal predictor data at the prediction time are used to estimate the landmark model at this time. (B) A dataset resembling the WisARD data where the longitudinal measurements are irregularly spaced and unsynchronized among study subjects. There is no predictor data at the prediction time, and data around the prediction time were assigned kernel weights and a kernel regression is applied to these adjacent data to estimate the landmark model at this time.

Figure S4: **Illustration of the longitudinal eGFR trajectories by baseline covariates.** We randomly selected WisARD patients for various combinations of baseline covariates (living donor: Y/N; having prior Tx: Y/N; hospitalization within 6 months after Tx: Y/N; rejection events within 6 months after Tx: Y/N). Minor smoothing has been applied to each trajectory to visualize the overall trend better and avoid clutter.

Section S2. Technical details of the statistical analysis

Figure S3 illustrates the landmark prediction framework in this paper. To simplify the discussion, we first

consider the situation where every subject follows the same clinical visits schedule (**Figure S3(A)**). Let $\widetilde{\mathrm{T}}$ be the time gap from the landmark time to graft failure or death, and ε be the event type indicator, which equals to 1 for graft failure, 2 for death, and 0 for censoring. Let C denote the time to censoring. The observed time to event is $T = min\{\widetilde{\mathrm{T}}, C\}$. Let $\boldsymbol{X}$ be the predictor variables at the landmark time. The goal of concurrent landmark prediction is to estimate the predicted probability of graft failure by the prediction horizon, $P(\widetilde{\mathrm{T}} \leq \Delta, \varepsilon = 1|\boldsymbol{X})$, the predicted probability of death by the prediction horizon, $P(\widetilde{\mathrm{T}} \leq \Delta, \varepsilon = 2|\boldsymbol{X})$, and the mean eGFR at the prediction horizon if the subject is alive without graft failure, denoted by $E(\mathrm{Y}(\Delta)|\boldsymbol{X}, \widetilde{\mathrm{T}} > \Delta)$. The first two probability can be obtained from cause-specific proportional hazard models for graft failure and death, by following the method in Wolbers et al (2009). To estimate the third, note that $E(\mathrm{Y}(\Delta)|\boldsymbol{X}, \widetilde{\mathrm{T}} > \Delta) = E(\mathrm{Y}(\Delta)|\boldsymbol{X}, \widetilde{\mathrm{T}} > \Delta, \mathrm{C} > \Delta) = E(\mathrm{Y}(\Delta)|\boldsymbol{X}, \mathrm{T} > \Delta)$. This derivation used the commonly made assumption in survival analysis that censoring is independent from the outcomes of the regression model. We model $E(\mathrm{Y}(\Delta)|\boldsymbol{X}, \mathrm{T} > \Delta)$ as a function of $\boldsymbol{X}$ and Δ using a GEE equation.

Here are the details of the cause-specific hazard models and the GEE model. The landmark dynamic prediction model included three sub-models. One was a cause-specific Cox proportional hazard (CSH) model for graft failure, treating death as censoring. The other one was a CSH model for death, treating graft failure as censoring. The use of a pair of Cox models, one of each "cause" of the terminal event (graft failure, death), is a widely used approach to competing risk prediction problem. The predicted probabilities for graft failure and death were calculated by combining the estimated cumulative hazard functions from both models in a closed form mathematical formula (Wolbers et al 2009). Of note, since the model applied to the at-risk patients by the landmark time, the outcome variables of these two Cox models were residual survival times, defined as the time gap between the landmark time and the end of follow-up. The third sub-model was a generalized estimating equations (GEE) model fitted to the longitudinal eGFR data measured between the prediction time and the prediction horizon. The model adjusted for the time gap between eGFR measurement time and landmark time to accommodate the time trend of eGFR data. The predicted eGFR at the horizon was calculated from the linear predictor of the model.

All three sub-models used time-invariant and time-varying predictors at the landmark time. Since not all recipients had an eGFR at the month of the landmark time, we used kernel weighting to borrow information from neighboring measurements. In the WisARD data, transplant recipients did not follow a common clinical visit schedule for eGFR and other clinical assessments, and the data are illustrated in **Figure S3(B)**. The predictor variables are not all available at the landmark time. To cope with this situation, we used the kernel weighting approach with local linear approximation, which was proposed in our previous work (Li et al 2017; Zhu et al 2019). The clinical visits close to the landmark time and their associated data were given a positive weight, with more weights on the data that are closer the landmark time. The cause-specific hazard model and GEE model can be estimated using kernel weighted data. All analysis were conducted in R 4.0.4. The CSH model at each landmark time was implemented using the coxph() function of the "survival" package. The landmark GEE model was implemented using "geepack" package, with working independence correlation structure.

Backward model selection was used to select a parsimonious model from the initial set of time-invariant and time-varying predictors. At each backward selection step, the predictor with the largest p-value was dropped until all remaining predictors had a significant p-value at the 0.05 level. We constrained the backward selection to always retain a small set of widely recognized important predictors during the model selection process, which include eGFR, patient age, indicator of living/deceased donor, and kidney donor profile index (KDPI). Beta-2 microglobulin (B2M) has a highly skewed distribution and was log-transformed before the analysis.

We fitted and evaluated the landmark models at 56 evenly spaced landmark times from post-transplantation month 6 to month 60. By confining the selected landmark times to 60 months, we ensured that there were enough outcome events during the data split of cross-validation. Nevertheless, for the sole purpose of model fitting, the landmark model could be estimated at any landmark time up to 120 months post-transplantation. Since the at-risk recipients and their data change slowly between consecutive months, the estimated parameters vary with

the landmark time as continuous curves. We performed backwards model selection at months 6, 12, 24, 36, 48 and 60. If a predictor passed model selection at any of these landmark times for at least one of the three sub-models, that predictor was included in the final landmark model at all the landmark times. For comparison, we fitted a static prediction model and compared it to the dynamic prediction results.

Section S3. An approximation to the measurement error variance of eGFR

Coresh et al (2002) reported the percentage of difference between two estimates of GFR among 1,919 NHANES III participants who had two measurements of serum creatinine a median of 17 days apart. Their Table 3 reported the summary statistics of the relative change in percentage difference. We used that data to calculate an approximated estimate of the variance of the absolute difference between the two eGFR values. Since these eGFRs were measured close in time, this variance can be taken as the measurement error variance of the eGFR, which reflects the physiological and bioassay variability and not any systematic change over time. This measurement error variance may be used as a lower bound for the prediction model of eGFR. Let $Y_1$and Y_2 be the two eGFR measurements, and define $D = Y_2 - Y_1$ be their difference. Table 3 of Coresh et al (2002) reported $var(D/Y_1)$, which can be written as

$$var\{E(D/Y_1\,|Y_1)\} + E\{var(D/Y_1\,|Y_1)\} = var\{E(D|Y_1)/Y_1\} + E\{var(D|Y_1)/Y_1^2\}.$$

Since $E(D|Y_1) \approx 0$, we can omit the first term. The presumption of this measurement error analysis is that the measurement error variance of eGFR is a constant and does not vary with the eGFR level. Then $var(D/Y_1) \approx E\{var(D)/Y_1^2\} \approx var(D)/E(Y_1^2)$. The latter approximation holds because $D \ll Y_1$. The $E(Y_1^2)$ can be calculated from the mean and variance of Y_1, which was reported in Table 3. By the procedure above, we estimate that the measurement error variance of eGFR, $var(D)$, is approximately 15.5 mL/min/1.73m^2. This quantity is close to the RMSE result in our **Figure 2**.

Section S4. Developing a parsimonious landmark model with reduced sets of predictors and comparing its prediction performance with the static model

The landmark model presented in the main text of the paper includes 21 predictors. These predictors are used in the cause-specific hazard (CSH) sub-model for graft failure, the CSH sub-model for death before graft failure, and the GEE sub-model for future eGFR. We have used a conservative variable selection rule that if a predictor is selected at a landmark time, it is selected for that sub-model; if a predictor is selected for a sub-model, it is retained for all sub-models. This conservative rule reduces the chance of missing important predictors and can be used to demonstrate that our proposed landmark analysis can accommodate relatively more time-invariant and time-varying predictors with tractable computation, in contrast to their joint modeling counterparts for dynamic prediction problems.

However, a weakness of this conservative approach is that it increases the number of predictors that a user must collect in order to use the prediction tool. If we can develop a more parsimonious model with fewer predictors but comparable prediction accuracy, that would be convenient from a practical perspective. In this section, we present a more parsimonious model from a less conservative model selection rule and study its prediction performance.

We started with a full model containing the 21 previously selected predictors. We removed the non-significant variables one at a time in a backward model selection procedure, using a p-value threshold of 0.05. This backward selection process was repeated at ten landmark times, from the 6th month to the 60th month post-transplantation. Variables significant at 50% or more of the landmark times were selected for each sub-model. We allowed each sub-model to have its own set of predictors and did not take the union of these three sets as the predictor set for the landmark model overall. The following is a description of the predictors for each sub-model.

- The CSH sub-model for graft failure has 7 predictors: eGFR at the landmark time, age at transplantation, whether the ESRD of the graft recipient was due to diabetes, any hospitalization or rejection event in the past 12 months, HLA mismatches, and whether the donor had diabetes.

- The CSH sub-model for death has 12 predictors: eGFR at the landmark time, age at transplantation of the graft recipient, whether the ESRD of the graft recipient was due to diabetes, any hospitalization or rejection event in the past 12 months, use of induction therapies (Alemtuzumab/IL2/ATG (Thymo)), use of CsA as a maintenance immunosuppressant, B2M, having prior transplantation, and pre-transplantation hemodialysis months.

- The GEE sub-model for future eGFR has 6 predictors: eGFR at the landmark time, any hospitalization or rejection event in the past 12 months, B2M, age of donor, and whether the donor had diabetes.

The cross-validated prediction performance of the reduced model is shown in **Figure S5** and **Figure S6**. By comparing Figure S5 to Figure 1 and comparing Figure S6 to Figure 2, the reduced model maintains approximately the same level of prediction accuracy as the 21-predictor model in the main text. In order to use this reduced model, the researcher needs to collect predictors in all three predictor sets, which amounts to 15 predictors. This is a notable reduction from the 21-predictor model.

The following are figure legends for Figures S5 and S6. High-resolution images of these figures are provided at the end of this document

Figure S5. The time-dependent AUC and BS of LM and SPM in predicting competing risks using the reduced set of predictors. The AUC and BS values vary with prediction time (ranging from 6 to 60 months post-transplant), and for three prediction horizons (1, 3, 5 years). The asterisk (∗) indicates that the difference in AUC or BS between LM and SPM is statistically significant as the 95% bootstrap confidence interval does not cover zero. Panel A: graft failure; Panel B: death.

Figure S6. Comparison of landmark GEE model with static GEE model in predicting the future eGFR using the reduced set of predictors. Predictive accuracy metrics for eGFR include the proportion that the predicted values fall within 30% (P30) and 50% (P50) of observed eGFR values at the prediction horizon, and the root mean squared error (RMSE, mL/min/1.73m^2) between the predicted and observed eGFR. The results are displayed by prediction horizon (1, 3, 5 years) and the prediction time ranges from 6 to 60 months post-transplant.

References

- Li L, Luo S, Hu B, Greene T. Dynamic Prediction of Renal Failure Using Longitudinal Biomarkers in a Cohort Study of Chronic Kidney Disease. *Statistics in Biosciences*. Dec 2017;9(2):357-378.
- Zhu YY, Li L, Huang XL. Landmark linear transformation model for dynamic prediction with application to a longitudinal cohort study of chronic disease. *Journal of the Royal Statistical Society Series C-Applied Statistics*. Apr 2019;68(3):771-791.
- Wolbers M, Koller MT, Witteman JC, Steyerberg EW. Prognostic models with competing risks: methods and application to coronary risk prediction. *Epidemiology*. Jul 2009;20(4):555-61.
- Coresh J, Astor BC, McQuillan G, et al. Calibration and random variation of the serum creatinine assay as critical elements of using equations to estimate glomerular filtration rate. *Am J Kidney Dis*. May 2002;39(5):920-9.

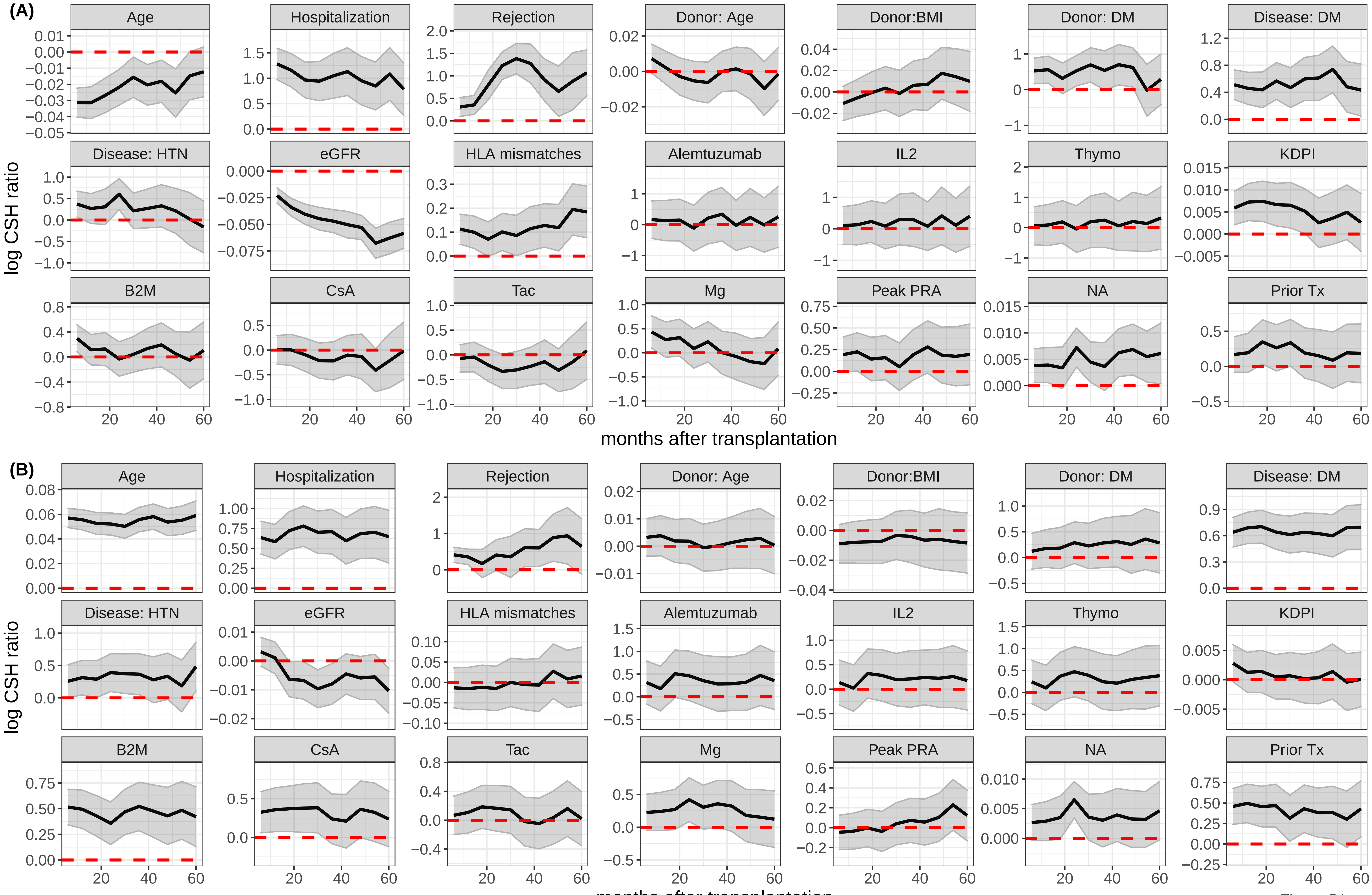
(A)
Age
Hospitalization
Rejection
Donor: Age
Donor:BMI
Donor: DM
Disease: DM
Disease: HTN
eGFR
HLA mismatches
Alemtuzumab
IL2
Thymo
KDPI
B2M
CsA
Tac
Mg
Peak PRA
NA
Prior Tx
log CSH ratio
months after transplantation
(B)
Age
Hospitalization
Rejection
Donor: Age
Donor:BMI
Donor: DM
Disease: DM
Disease: HTN
eGFR
HLA mismatches
Alemtuzumab
IL2
Thymo
KDPI
B2M
CsA
Tac
Mg
Peak PRA
NA
Prior Tx
log CSH ratio
months after transplantation

Figure S1

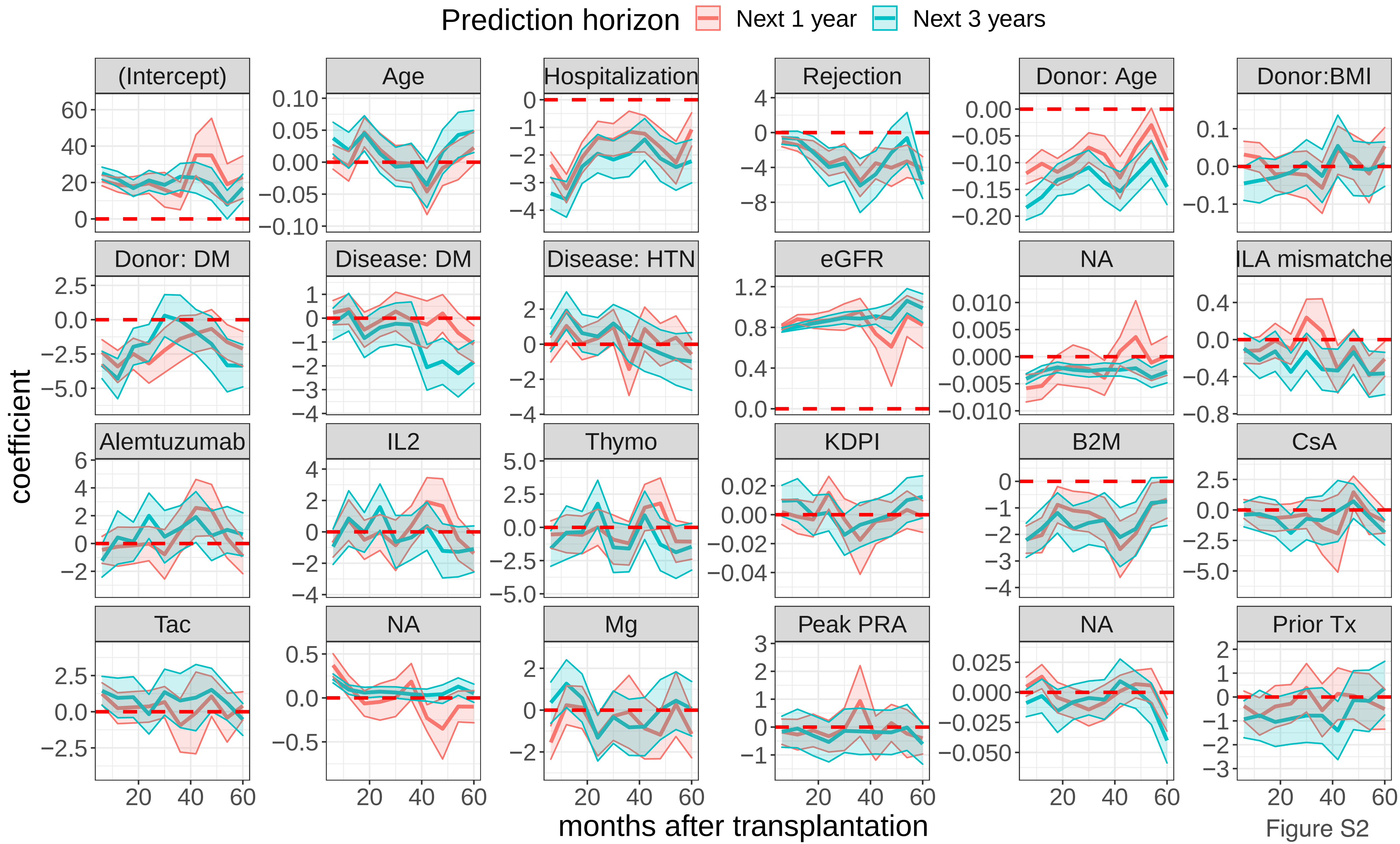
Prediction horizon
Next 1 year
Next 3 years
(Intercept)
Age
Hospitalization
Rejection
Donor: Age
Donor:BMI
Donor: DM
Disease: DM
Disease: HTN
eGFR
NA
ILA mismatche
Alemtuzumab
IL2
Thymo
KDPI
B2M
CsA
Tac
NA
Mg
Peak PRA
NA
Prior Tx
coefficient
months after transplantation

Figure S2

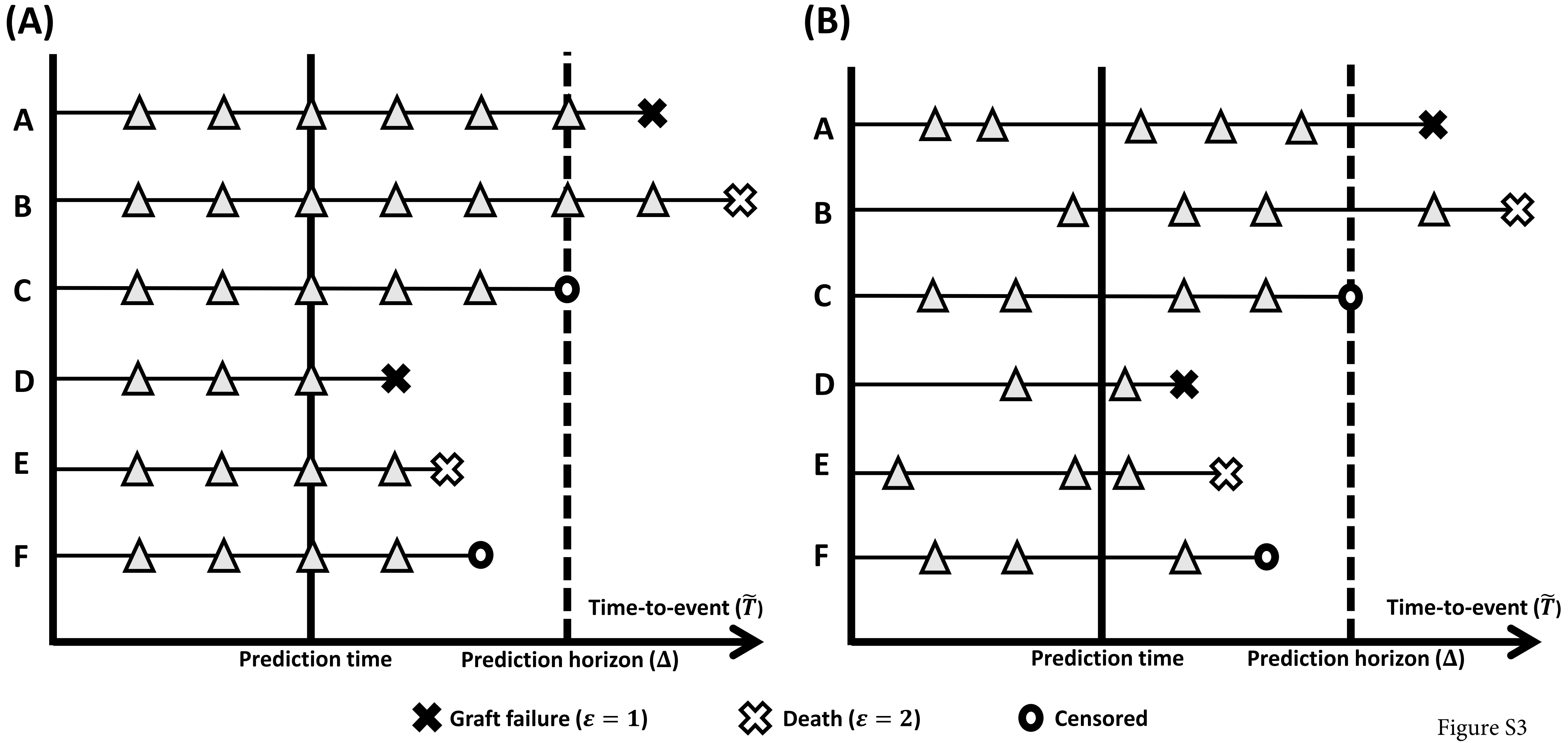
(A)
A
B
C
D
E
F
Time-to-event ($\widetilde{T}$)
Prediction time
Prediction horizon (Δ)
(B)
A
B
C
D
E
F
Time-to-event ($\widetilde{T}$)
Prediction time
Prediction horizon (Δ)
Graft failure ($\varepsilon = 1$)
Death ($\varepsilon = 2$)
Censored

Figure S3

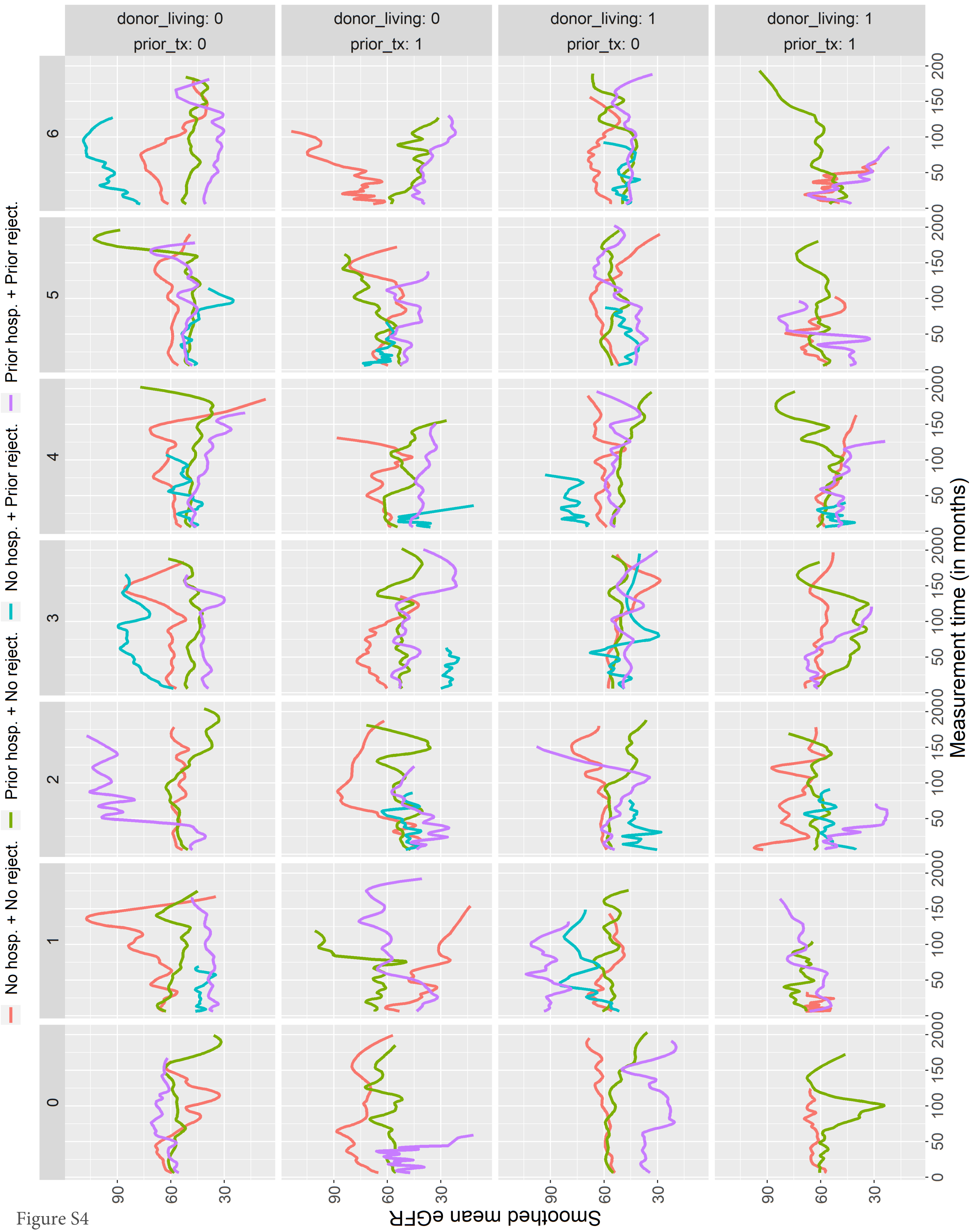

No hosp. + No reject.
Prior hosp. + No reject.
No hosp. + Prior reject.
Prior hosp. + Prior reject.
donor_living: 0
prior_tx: 0
donor_living: 0
prior_tx: 1
donor_living: 1
prior_tx: 0
donor_living: 1
prior_tx: 1
0
1
2
3
4
5
6
90
60
30
0
50
100
150
200
Smoothed mean eGFR
Measurement time (in months)

Figure S4

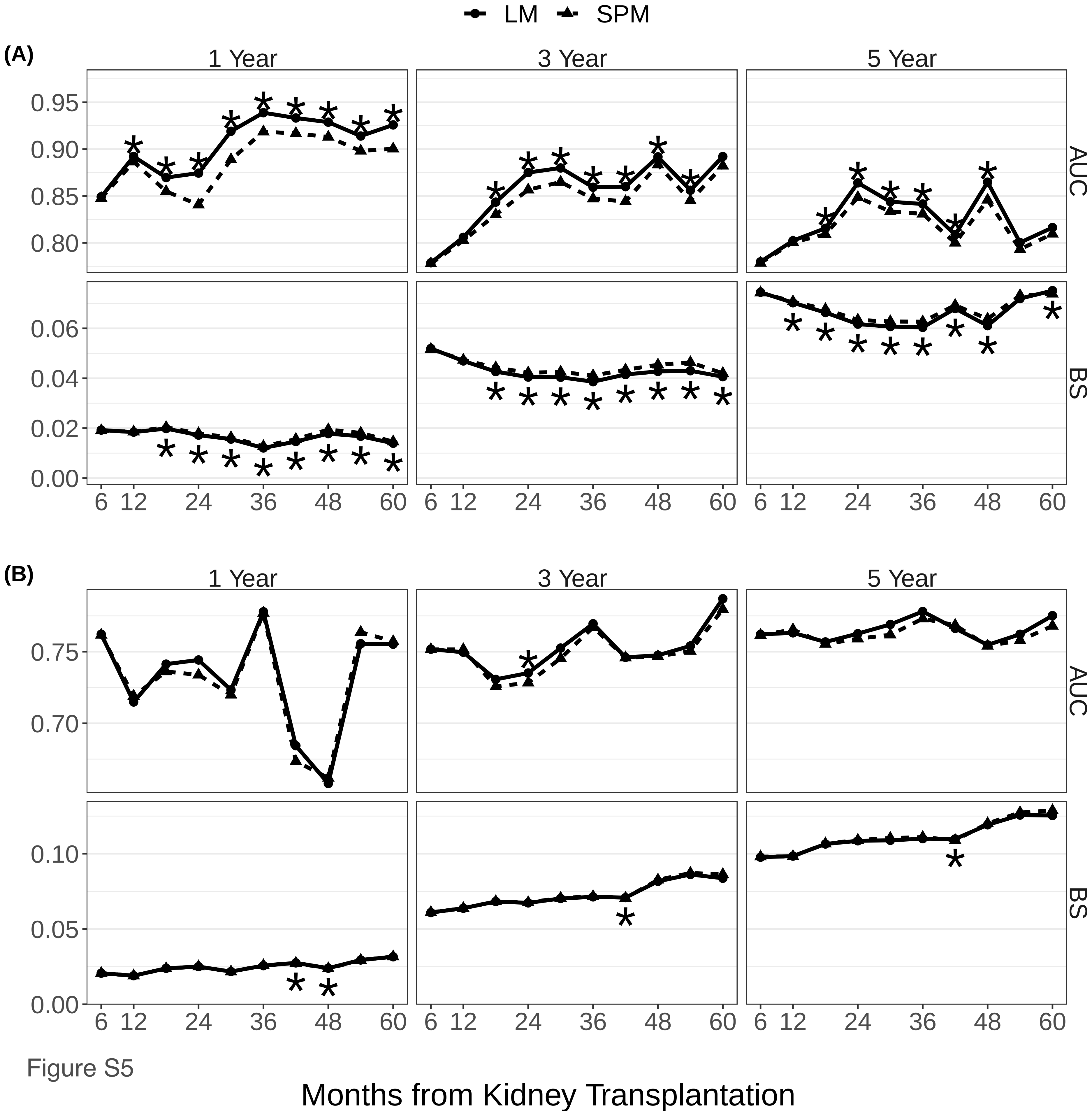
LM
SPM
(A)
1 Year
3 Year
5 Year
AUC
BS
0.95
0.90
0.85
0.80
0.06
0.04
0.02
0.00
6 12 24 36 48 60
(B)
1 Year
3 Year
5 Year
AUC
BS
0.75
0.70
0.10
0.05
0.00
Months from Kidney Transplantation

Figure S5

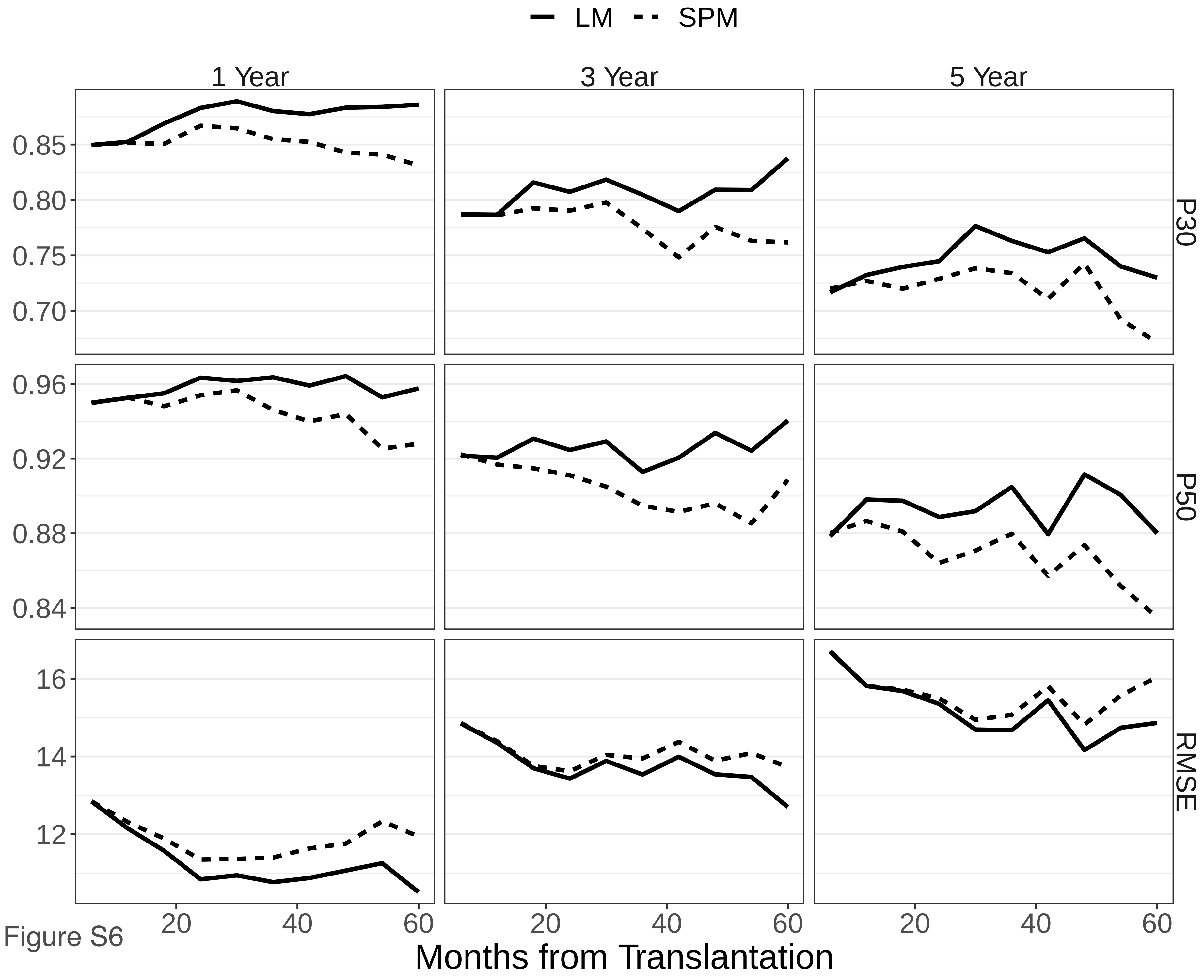

LM
SPM
1 Year
3 Year
5 Year
P30
P50
RMSE
0.85
0.80
0.75
0.70
0.96
0.92
0.88
0.84
16
14
12
20
40
60
Months from Translantation

Figure S6
